# Endoscopic Ultrasound-Guided Versus Percutaneous Transhepatic Biliary Drainage After Failed Endoscopic Retrograde Cholangiopancreatography in Malignant Biliary Obstruction: A Single-Center Retrospective Cohort

**DOI:** 10.3390/cancers18050783

**Published:** 2026-02-28

**Authors:** Wojciech Ciesielski, Łukasz Durko, Ludomir Stefańczyk, Adam Dobek, Anna Bulicz, Amelia Wojnicka, Zuzanna Sosnowska, Agata Grochowska, Janusz Strzelczyk, Piotr Hogendorf, Adam Durczyński, Tomasz Klimczak

**Affiliations:** 1 Department of General and Transplant Surgery, Medical University of Łódź, 90-153 Lodz, Poland; wojciech.ciesielski@umed.lodz.pl (W.C.); anna.bulicz@stud.umed.lodz.pl (A.B.); amelia.wojnicka@stud.umed.lodz.pl (A.W.); zuzanna.sosnowska@stud.umed.lodz.pl (Z.S.); agata.grochowska@stud.umed.lodz.pl (A.G.); janusz.strzelczyk@umed.lodz.pl (J.S.); piotr.hogendorf@umed.lodz.pl (P.H.); adam.durczynski@umed.lodz.pl (A.D.); 2 Department of Digestive Tract Diseases, Medical University of Łódź, 90-153 Lodz, Poland; lukasz.durko@umed.lodz.pl; 3 1st Department of Radiology and Diagnostic Imaging, Medical University of Łódź, 90-153 Lodz, Poland; ludomir.stefanczyk@umed.lodz.pl (L.S.); adam.dobek@umed.lodz.pl (A.D.)

**Keywords:** malignant biliary obstruction, endoscopic ultrasound-guided biliary drainage, percutaneous transhepatic biliary drainage, ERCP failure

## Abstract

**Background:** After a failed endoscopic retrograde cholangiopancreatography (ERCP) for malignant biliary obstruction (MBO), second-line drainage is performed with endoscopic ultrasound-guided biliary drainage (EUS-BD) or percutaneous transhepatic biliary drainage (PTBD). We compared their effectiveness, safety, and short-term survival. **Methods:** We conducted a single-center retrospective cohort of 101 adults with MBO after they had experienced a failed ERCP (EUS-BD *n* = 37; PTBD *n* = 64). Allocation was non-randomized and driven by operational availability. Baseline laboratory tests (complete blood count, platelets, and C-reactive protein) and derived indices (neutrophil-to-lymphocyte ratio [NLR], platelet-to-lymphocyte ratio [PLR], lymphocyte-to-monocyte ratio [LMR], systemic immune-inflammation index [SII], systemic inflammation response index [SIRI], neutrophil-to-platelet score [NPS], and lymphocyte-to-CRP ratio [LCR]) were compared. Outcomes that were a technical success include: an early biochemical response (bilirubin reduction), complications (Clavien–Dindo), length of stay (LOS), and overall survival (OS). Between-group comparisons used the two-sided Mann–Whitney U test (continuous) and Fisher’s exact (binary) test. Survival was assessed by the Kaplan–Meier estimator using log-rank testing. To address later adoption of EUS-BD, we also estimated a restricted mean survival time of 180 days (RMST_0–180) with 95% confidence intervals (CIs). **Results:** Baseline inflammatory markers and composite indices were similar; baseline total bilirubin was higher in PTBD. The technical success was 100% in both groups. Early biochemical response was 86.5% after EUS-BD vs. 78.1% after PTBD (*p* = 0.43). Any complication occurred in 29.7% vs. 12.5% (*p* = 0.04); major complications (Clavien–Dindo ≥ III) occurred in 10.8% vs. 0% (*p* = 0.02), respectively; and the LOS did not differ (*p* = 0.21). OS favored EUS-BD (median 143 vs. 54 days and log-rank *p* = 0.012). RMST_0–180 was 111.1 days for EUS-BD vs. 71.4 days for PTBD (difference + 39.6 days; 95% CI 11.3–65.9). **Conclusions:** After a failed ERCP for MBO, EUS-BD and PTBD achieved universal technical success and similar early biochemical responses, but EUS-BD was associated with higher complication rates and a significantly longer six-month survival. These findings support the individualized selection balancing procedural risk with the anticipated survival benefit and highlight the need for prospective comparative studies.

## Simple Summary

Malignant biliary obstruction (MBO) can be managed through endoscopic retrograde cholangiopancreatography (ERCP) with stent placement. However, in less than 10% of cases, this method fails to restore biliary flow. In such cases, endoscopic ultrasound biliary drainage methods (EUS-BD) and percutaneous transhepatic biliary drainage (PTBD) can be used as an alternative approach. It remains unclear which procedure offers better outcomes for patients. In this study of 101 patients with MBO after a failed ERCP, we compared these two methods in terms of effectiveness, safety, and survival. Both techniques successfully relieved bile duct obstruction, but they differed in complication rates and patient survival. Our findings may help clinicians select the most appropriate drainage method and inform future research aimed at improving care for patients with advanced biliary cancers.

## 1. Introduction

The most common cause of extrahepatic jaundice is choledocholithiasis, followed by hepatobiliopancreatic malignant tumors such as pancreatic cancer (especially located in the head) or bile duct cancer. Less common causes include bile duct strictures and acute or chronic pancreatitis [1]. The primary focus of our study is malignant biliary obstruction (MBO).

MBO is usually managed through endoscopic retrograde cholangiopancreatography (ERCP), which allows a biliary stent to be placed in the bile duct to provide sufficient drainage, resulting in a rapid improvement in liver function and in the patient’s clinical condition. In over 90% of MBO, this method is successful in restoring proper bile duct patency. In less than 10% of failed ERCP cases, the use of alternative methods may be necessary [2,3].

As alternatives, endoscopic ultrasound-guided biliary drainage (EUS-BD) or percutaneous transhepatic biliary drainage (PTBD) methods can be implemented. EUS-BD allows for better visualization of the site of biliary obstruction and better access to the bile duct through the gastrointestinal tract [4]. For EUS-BD, a few techniques have been distinguished based on whether the access point is intra- or extrahepatic and how the stent is being placed (transduodenal or transgastric approach) [5]. Among the various techniques for restoring biliary patency, EUS-choledochoduodenostomy (CDS), EUS-hepaticogastrostomy (HGS), and EUS-gallbladder drainage (GBD) can be distinguished.

PTBD involves inserting the tip of a catheter into the bile ducts under ultrasound or fluoroscopy guidance and allowing bile to drain extracorporeally [6]. In this technique the left hepatic duct (LHD), the right hepatic duct (RHD), the gallbladder (GB), and the common bile duct (CBD) can be drained. As in EUS-BD, the choice of drainage site is indicated by the technical capabilities of the drainage, the anatomy of the bile ducts (sufficient widening of biliary ducts, usually above 8 mm for segmental/lobular intrahepatic bile duct dilatation), and the location of the tumor [7].

Biliary drainage can reduce plasma bilirubin concentrations and mitigate the clinical manifestations of jaundice, improving patients’ quality of life and optimizing their physiological status for subsequent surgical resection, neoadjuvant systemic therapy, or, like in our case, palliative chemotherapy.

This study aims to compare EUS-BD and PTBD in terms of the risk, effectiveness, and safety in patients with MBO. Also, overall survival (OS) of patients with MBO was analyzed accordingly.

## 2. Materials and Methods

We conducted a single-center, retrospective cohort study of 101 consecutive adults with distal and proximal MBO in whom ERCP failed in a high-volume Hepato-Pancreato-Biliary (HPB) unit. A failed ERCP was defined as an unsuccessful biliary cannulation, thus was unable to achieve adequate biliary drainage despite the use of standard and advanced endoscopic techniques. After ERCP failure, patients were evaluated for EUS-BD feasibility based on anatomical conditions and clinical status; PTBD was selected when EUS-BD was not technically feasible and not immediately available.

Patients subsequently underwent either EUS-BD or PTBD. Allocation was non-randomized and determined solely by the operational availability of equipment and trained personnel at the time of clinical decision-making. There was no a priori clinician or patient preference between modalities. This study was conducted in accordance with the ethical standards of the institutional and national research committee and the 1964 Helsinki Declaration and its amendments. Institutional Review Board (IRB) approval was waived due to the retrospective nature of the study. The design of the study is based on retrospective analysis of fully anonymized clinical data with no further interventions.

Because EUS-BD was implemented later at our center, the study periods differed between groups. PTBD cases were completed from 31 July 2020 to 8 March 2025. EUS-BD was introduced in Q4 2023, and EUS cases were collected from 12 October 2023 to 27 March 2025. Both procedures were performed by experienced operators: interventional radiologists for PTBD and interventional endoscopists for EUS-BD.

### 2.1. PTBD Technique

**Figure 1:**
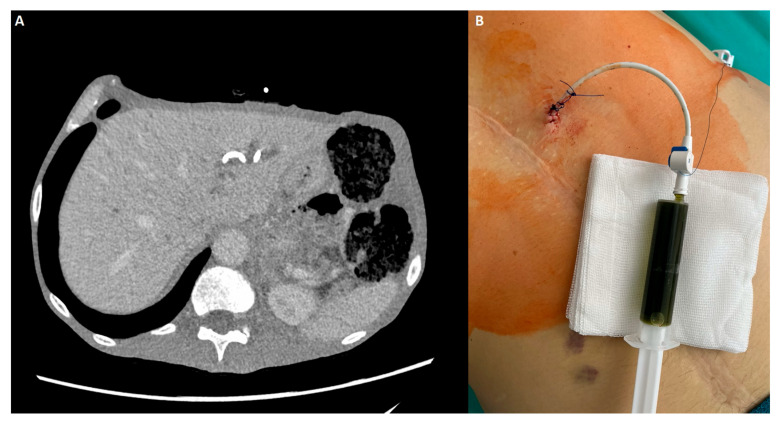
Percutaneous transhepatic biliary drain (PTBD) in the left hepatic duct (LHD) in patient with malignant biliary obstruction (MBO) after a failed endoscopic retrograde cholangiopancreatography (ERCP). (**A**) Computed tomography (CT) presenting a pigtail in the lumen of the biliary tract. (**B**) External drain with bile in the syringe collected straight after the procedure.

**Figure 2:**
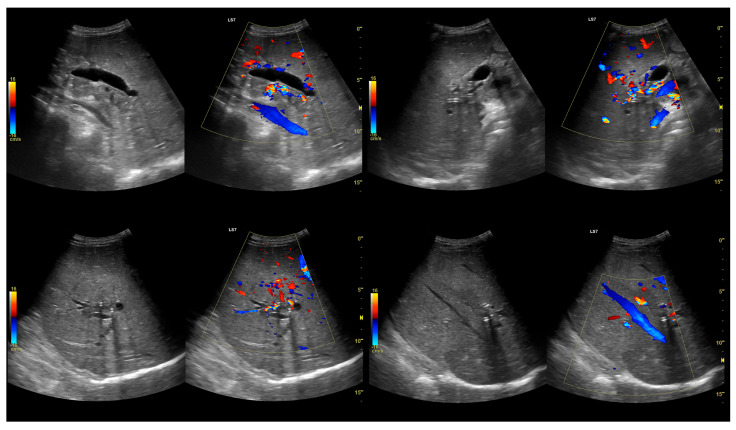
Percutaneous transhepatic biliary drain (PTBD) in the right hepatic duct (RHD) in a patient with malignant biliary obstruction (MBO) after a failed endoscopic retrograde cholangiopancreatography (ERCP).

**Figure 3:**
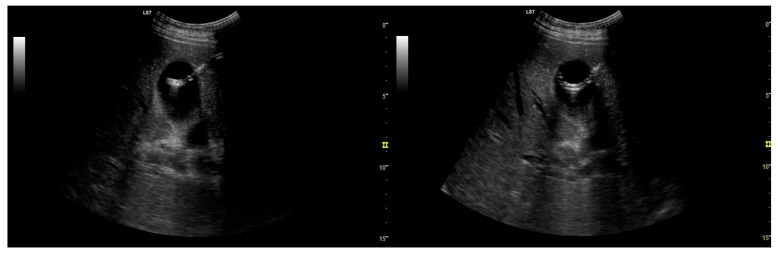
Percutaneous transhepatic biliary drain (PTBD) in the gallbladder (GB) in patient with malignant biliary obstruction (MBO) after a failed endoscopic retrograde cholangiopancreatography (ERCP).

All percutaneous procedures were performed by a board-certified interventional radiologist (L.S. assisted by W.C.) trained in image-guided hepatobiliary access, peri-procedural care, and complications management. Under anesthesiologist-led sedation and local infiltration with 1% lidocaine, patients were positioned supine. Ultrasound guidance was provided by a LOGIQ 7 system (GE Healthcare, Milwaukee, WI, USA) with a 4C convex transducer. External biliary drainage (not stenting) used Ultra-Pro II disposable needle guides (CIVCO Medical Solutions, Coralville, IA, USA) and 8.5 F or 10 F ReSolve Biliary Locking Drainage Catheters (Merit Medical, South Jordan, UT, USA) in the left hepatic duct (LHD) (Figure 1), the right hepatic duct (RHD) (Figure 2), the gall bladder (GB) (Figure 3), or the common bile duct (CBD). No percutaneous metallic stents were used during the study period due to local availability and training constraints. Scheduled internalization to SEMS was not part of the institutional protocol at the time.

### 2.2. EUS-BD Technique

**Figure 4:**
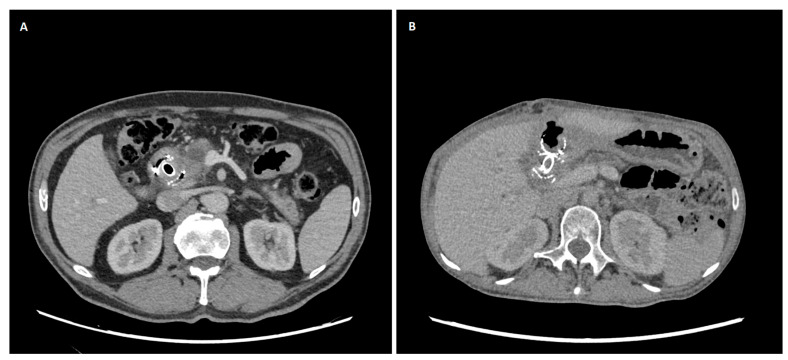
Computed tomography (CT) scans of patients who underwent endoscopic ultrasound-guided biliary drainage due to malignant biliary obstruction (MBO). (**A**) Cholecystogastrostomy. (**B**) Choledochoduodenostomy.

**Figure 5:**
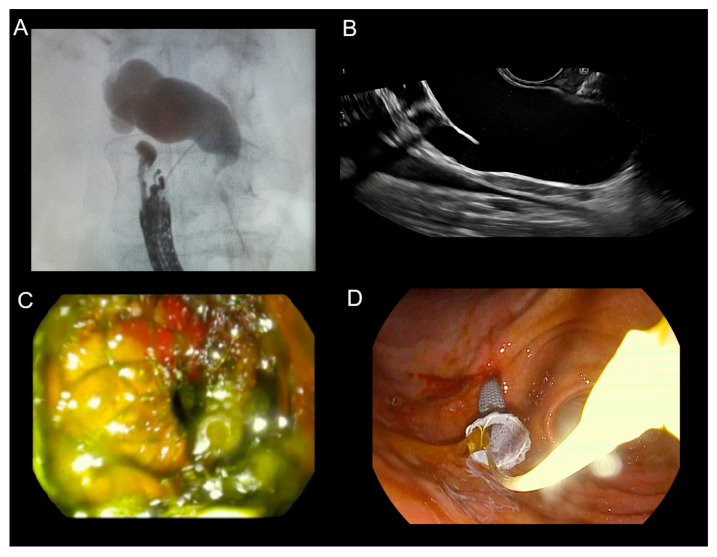
Endoscopic ultrasound-guided biliary drainage (EUS-BD) technique. (**A**) Fluoroscopic cholangiogram that is demonstrating a dilated common bile duct (CBD). (**B**) EUS view of the dilated bile duct with the puncture needle in situ. (**C**) Endoscopic view of a choledochoduodenostomy with a fully expanded Hot AXIOS lumen-apposing metal stent (LAMS). (**D**) Endoscopic view of a hepaticogastrostomy with a fully expanded Giobor LAMS.

In cases that proceeded to use EUS-guided biliary drainage, the same endoscopist who performed the failed ERCP also performed the subsequent EUS-BD procedure, ensuring continuity of care and demonstrating sufficient operator’s skills and expertise (T.K. and Ł.D.). All EUS-BD procedures were performed by interventional endoscopists with documented competency in therapeutic EUS and ERCP, in accordance with contemporary ESGE/ASGE guidance for training and quality standards in interventional EUS (completion of a structured EUS curriculum, competence in diagnostic EUS, substantial ERCP experience, and supervised progression to EUS-guided biliary drainage). Under general anesthesia, patients were positioned supine or in the left lateral decubitus position. Electrocautery-enhanced lumen-apposing metal stents (ECE-LAMS), Hot AXIOS (Boston Scientific, Marlborough, MA, USA), Giobor (Taewoong Medical, Gimpo-si, Republic of Korea), or Hot SPAXUS (Taewoong Medical, Gimpo-si, Republic of Korea) were deployed using a therapeutic linear echoendoscope (PENTAX EG38-J10UT, PENTAX Europe GmbH, Hamburg, Germany) to create EUS-CDS, EUS-HGS, or EUS-GBD as anatomically appropriate (Figure 4 and Figure 5). EUS-CDS was the preferred approach in cases of distal MBO and was performed whenever a safe transduodenal window and an adequate common bile duct dilation were present. EUS-HGS was selected in patients with proximal MBO, duodenal obstruction or inaccessibility, papillary involvement, or altered postoperative anatomy, precluding EUS-CDS. EUS-CGS was used as a salvage technique in highly selected cases when both EUS-CDS and EUS-HGS were technically unfeasible.

EUS-GBD gallbladder drainage was used as a biliary decompression strategy in selected patients with distal malignant obstruction. Cystic duct patency was an anticipated pre-procedure by review of imaging (level of obstruction in the common bile duct and gallbladder enlargement) and confirmed intra-procedurally under fluoroscopy. If opacification suggested cystic duct obstruction, EUS-GBD was not pursued.

Baseline demographics (age and sex), procedure type and subtype (EUS: CDS/HGS/GBD and PTBD: LHD/RHD/GB/CBD), pre-procedure laboratory tests (complete blood count, platelets, and C-reactive protein (CRP)), and derived inflammatory indices (neutrophil-to-lymphocyte ratio (NLR), platelet-to-lymphocyte ratio (PLR), lymphocyte-to-monocyte ratio (LMR), systemic immune-inflammation index (SII), systemic inflammation response index (SIRI), neutrophil-platelet score (NPS), and lymphocyte-C-reactive protein ratio (LCR)) were recorded.

Post-procedural data included the length of stay (LOS), adverse events (AE) graded by the Clavien–Dindo scale, transfusion requirements, technical success (correct device placement with immediate decompression), early biochemical response (≥5% reduction in total bilirubin by post-procedure day 2), and overall survival (OS).

Because patients were typically discharged shortly after drainage and continued care in oncology clinics, routine day-14 laboratory follow-up was not uniformly available. We therefore prespecified an early biochemical response as ≥5% reduction in total bilirubin by post-procedure day 2 (D+2), chosen to reflect discharge readiness and early effectiveness. In recognition of guideline definitions that often use ≥50% reduction or normalization by day 14, we refer to our D+2 metric as “early biochemical response (day-2)” throughout.

AEs were identified through review of procedure notes, nursing records, laboratory trends, imaging, and discharge summaries. Attribution to the biliary drainage procedure was adjudicated by two investigators based on temporal relationship and plausibility; discrepancies were resolved by consensus. When multiple AEs occurred in one patient, all events were listed, and the worst Clavien–Dindo grade was used for the “major complication” endpoint (≥III). AE definitions:

- Bleeding: any of the following within 7 days: intraprocedural hemorrhage requiring hemostasis; Hb drop ≥ 2.0 g/dL and clinical concern; transfusion; or intervention (endoscopic, radiologic, or surgical). Hemobilia was classified as bleeding if it met these criteria.
- Bile leak/biloma/bile peritonitis: bilious output from access tract or peritoneal signs plus a confirmation on imaging (US/CT) or need for drainage/intervention.
- Perforation/viscus injury: extraluminal air/contrast with peritoneal signs or need for endoscopic/radiologic/surgical management.
- Stent/drain maldeployment or migration: failure of intended position, need for rescue device, or secondary procedure for repositioning/replacement. (For EUS-BD: LAMS misdeployment and for PTBD: catheter malposition/dislodgement, tract loss).
- Cholangitis: fever ≥ 38.0 °C with cholestasis and systemic/inflammatory response requiring antibiotics (operationalized per Tokyo Guidelines criteria and adapted to post-drainage setting).
- Sepsis: infection with organ dysfunction consistent with Sepsis-3 (Sequential Organ Failure Assessment SOFA rise ≥ 2) requiring vasopressors or intensive care unit (ICU).
- Acute pancreatitis: new abdominal pain plus amylase/lipase ≥ 3× upper limit and/or imaging.
- Pneumoperitoneum/pneumothorax/subcutaneous emphysema: if symptomatic or requiring intervention (otherwise recorded as incidental).
- Access-site issues: pericatheter leakage causing skin breakdown/infection, tract infection/abscess, or need for unplanned exchange.
- Aspiration/event related to anesthesia: documented by anesthesiology and requiring treatment.

The composite index “any complication” included all prespecified AEs (any grade). “Major complication” was defined as Clavien–Dindo ≥ III (requiring endoscopic, radiologic, or surgical intervention; ICU; or causing persistent disability/death). We report AE rates (*n*/N, %) by treatment arm and subtype and provide exact 95% Clopper–Pearson CIs for key proportions.

### 2.3. Statistical Analysis

Continuous variables are reported as median (interquartile range, IQR) and categorical variables as *n* (%). Between-group comparisons (EUS-BD vs. PTBD) for continuous measures (e.g., CRP, platelets, and inflammatory indices) used the two-sided Mann–Whitney U test; nominal counts (e.g., sex) are presented descriptively. For procedural outcomes, overall EUS–PTBD comparisons used Fisher’s exact test for binary endpoints (early biochemical response ≥ 5% bilirubin reduction by day 2; any complication; and major complication, Clavien–Dindo ≥ III) and Mann–Whitney U test for the LOS. Within-method subtype analyses (EUS: GBD/CDS/HGS and PTBD: LHD/RHD/GB/CBD) used the chi-square test or Fisher’s exact test for binary variables and Kruskal–Wallis test for the LOS.

The OS was measured from the index procedure to death from any cause. All deaths were cancer related. Patients whose cause of death was not cancer-related were excluded from further analysis. Survivors were censored on 1 September 2025. Survival functions were estimated with Kaplan–Meier curves and compared with the two-sided log-rank test. Given the later adoption of EUS-BD (non-overlapping follow-up windows among survivors), we additionally quantified average survival within a common horizon using a restricted mean survival time of τ = 180 days (RMST_0–180_), reported for each arm. RMST represents the area under the KM curve on 0, τ and is interpretable as the average survival time accumulated within the first τ days. We chose τ = 180 days a priori to balance clinical relevance and statistical stability. At this horizon, the proportion remaining under observation (“at risk”) was 21.6% (8/37) for EUS and 14.1% (9/64) for PTBD, satisfying the practical guideline that ≥10–20% of patients remain at risk in each arm at τ. RMST and the between-group difference (EUS–PTBD) were reported with 95% confidence intervals. Number-at-risk counts were tabulated at 0, 30, 90, 180, and 360 days to document follow-up density.

All tests were two-sided with α = 0.05; *p* < 0.05 was considered statistically significant. Exact *p*-values and effect sizes with 95% confidence intervals accompany all comparisons.

## 3. Results

We analyzed 101 consecutive patients (EUS-BD, *n* = 37 and PTBD, *n* = 64) after a failed ERCP. Almost all cases of MBO were caused by pancreatic head and periampullary cancer, accounting for 80% of analyzed cases. Proximal obstruction was, in general, associated with unresectable cholangiocarcinoma or metastatic adenocarcinoma. All ERCP procedures were performed by experienced interventional endoscopists. Baseline inflammatory markers and composite indices (NLR, PLR, LMR, SII, SIRI, NPS, and LCR) did not differ significantly between groups, whereas baseline total bilirubin was higher in the PTBD cohort (Table 1). Detailed medians with IQRs and two-sided Mann–Whitney *p*-values are provided in Table 1; categorical distributions are shown descriptively.

Technical success was achieved at 100% in both arms (no between-group test applicable). Early biochemical response (≥5% bilirubin reduction by day two) was 86.5% (32/37) for EUS and 78.1% (50/64) for PTBD (Fisher, *p* = 0.43). Any complication occurred more often after EUS (29.7%, 11/37) than after PTBD (12.5%, 8/64 and Fisher, *p* = 0.04). Major complications (Clavien–Dindo ≥ III) were observed in 10.8% (4/37) after EUS and 0% (0/64) after PTBD (Fisher, *p* = 0.02). The LOS was similar between the groups, and the difference was not statistically significant (EUS 4.0 [2.0–7.0] vs. PTBD 3.5 [2.0–6.0] days and Mann–Whitney, *p* = 0.21). Within-method subtype analyses were exploratory: in EUS subtypes, early biochemical response differed significantly (GBD 66.7%, CDS 100.0%, HGS 70.0% and *p* = 0.02), and any complication varied across subtypes (*p* = 0.049), while the LOS did not (*p* = 0.28). In PTBD subtypes, neither early biochemical response (*p* = 0.38), any complication (*p* = 0.17), nor the LOS (*p* = 0.15) differed significantly. Major complications were absent in all PTBD subgroups, precluding formal testing. Procedural outcomes are summarized in Table 2.

AEs are summarized by category and typical management in Table 3. In the EUS arm, 11/37 (29.7%) patients experienced at least one AE. The most frequent category was bleeding/anemisation (8/37 and 18.9%). We also observed one fluid collection that was consistent with a biloma (1/37 and 2.7%) and two gastrointestinal perforations (2/37 and 5.4%)—one was caused by stent migration, and one was caused by the rupture of the duodenal lesion; two patients died due to the severity of the AEs (2/37 and 5.4%). The worst recorded severity in the EUS cohort was Clavien–Dindo II in 7/37 (18.9%), IIIa in 2/37 (5.4%), and V in 2/37 (5.4%). As per the classification, grade II events were managed conservatively (including blood transfusion), whereas grade IIIa required endoscopic or radiologic intervention without general anesthesia and grade V reflect the deaths coded in the dataset as complications.

In the PTBD arm, AEs occurred in 8/64 (12.5%) and all were recorded as hemobilia or periliver hematoma with Clavien–Dindo II severity; no ≥III events or deaths were attributed as procedure-related complications in this group. Management comprised conservative measures and/or transfusion; drain revision was performed when clinically indicated.

**Figure 6:**
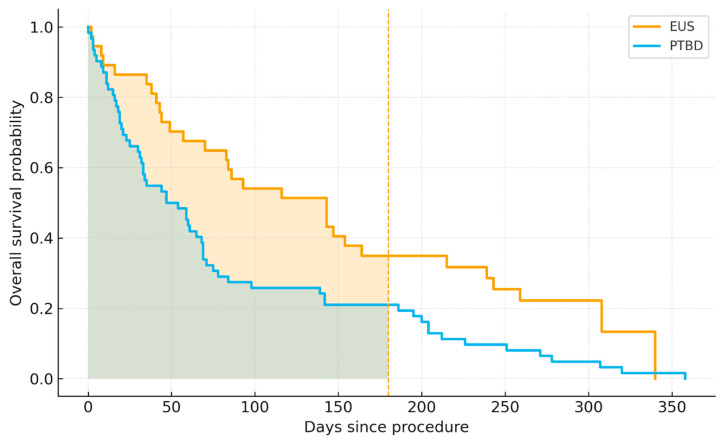
Kaplan–Meier overall survival after EUS-guided biliary drainage (EUS-BD) versus percutaneous transhepatic biliary drainage (PTBD). Median OS: EUS 143 days vs. PTBD 54 days and log-rank *p* = 0.012. For a horizon-matched comparison, restricted mean survival time of 180 days marked as the dashed line (RMST_0–180) was 111.1 days for EUS (95% CI 89.4–131.3) and 71.4 days for PTBD (95% CI 55.4–87.6), yielding a difference of 39.6 days (95% CI 11.3–65.9) in favor of EUS. Abbreviations: EUS-BD—endoscopic ultrasound-guided biliary drainage; PTBD—percutaneous transhepatic biliary drainage; OS—overall survival; RMST—restricted mean survival time; and CI—confidence interval.

Kaplan–Meier analysis demonstrated significantly longer OS with EUS compared to PTBD (median 143 vs. 54 days and log-rank *p* = 0.012). Over a common 180-day horizon, restricted mean survival time was 111.1 days after EUS versus 71.4 days after PTBD (difference of 39.6 days and 95% CI 11.3–65.9), indicating that patients treated with EUS accrued approximately 40 additional days of survival within the first six months (Figure 6).

Numbers at risk at 0/30/90/180/360 days were EUS-BD: 37/26/15/8/2 and PTBD: 62/41/19/8/2 (Table 4). At τ = 180, the proportions remaining under observation were 21.6% (8/37) for EUS and 12.9% (8/62) for PTBD, satisfying the ≥10–20% heuristic supporting the chosen horizon.

The numbers-at-risk table summarizes how many patients in each arm remained under observation (alive and uncensored) at prespecified landmarks along the Kaplan–Meier curves. At day zero, risk sets reflect the initial cohort sizes (EUS-BD 37, PTBD 64). Progressive attrition is seen at 30 and 90 days (EUS-BD 26/15, PTBD 42/20), and by 180 days both groups have eight patients at risk, indicating comparable follow-up density at the primary horizon used for RMST. By 360 days, only two patients remain at risk in each arm, highlighting substantial statistical uncertainty in the far tail of the curves and cautioning against over-interpreting late survival estimates. Clinically, these counts support two key inferences: (i) the choice of τ = 180 days is appropriate because ≥10–20% of each arm remains under observation at that landmark (EUS-BD 21.6%, PTBD 12.5%), and (ii) the observed early survival advantage of EUS-BD (higher median OS and larger RMST_0–180_) is not driven by asymmetric censoring but is consistent with a greater accumulation of survival time within the first six months. Conversely, beyond six months, the very small risk sets imply that differences should be interpreted cautiously, and RMST_0–180_ provides a more robust, clinically interpretable summary than late-time KM estimates.

## 4. Discussion

### 4.1. Summary of the Results

In this high-volume, single-center study of 101 patients with MBO after a failed ERCP, both methods, EUS-BD and PTBD, were found to be effective in terms of reaching technical success, confirming their feasibility when used by experienced operators. The early bilirubin level drop on day two was similar for both techniques (86.5% for EUS-BD and 78.1% for PTBD). Even though the effectiveness was comparable, EUS-BD was associated with a significantly greater risk of complications (29.7% vs. 12.5%), and, importantly, major complications (Clavien–Dindo ≥ III) occurred exclusively after EUS-BD (10.8% vs. 0%). The LOS was generally short and similar between groups.

Subgroup analysis of EUS-BD revealed high heterogeneity: EUS-CDS achieved early biochemical response of 100%, whereas EUS-GBD and EUS-HGS demonstrated lower response rates (66.7% and 70%, respectively) and higher complication frequencies, especially in the EUS-HGS subgroup (50% had Clavien–Dindo ≥ III complication). In contrast, PTBD subtypes exhibited no significant differences in major complication rates or the LOS.

The analysis of survival demonstrated a longer observed overall survival in patients who underwent EUS-BD compared with those treated with PTBD (median 143 vs. 54 days). Over 180 days, restricted mean survival time favored EUS-BD by approximately 40 days, which indicates that patients treated with EUS-BD accumulated more survival time during the first six months after drainage.

Overall, our findings suggest an association between EUS-BD and longer observed overall survival, while early biliary decompression rates were comparable between EUS-BD and PTBD. However, EUS-BD is burdened by a higher risk of complications, including serious ones, especially when more complex EUS-BD procedures are required. These observations should be interpreted cautiously given the retrospective design and non-randomized allocation of treatment modalities.

### 4.2. Literature Review

Meta-analysis by Zafar et al. included 668 patients across eight randomized trials of EUS-BD with ERCP or PTBD when treating MBO. The meta-analysis reported no significant differences in technical or early biochemical response between the two techniques. In contrast to our cohort, where EUS-BD was associated with a higher rate of complications, Zafar et al. found that EUS-BD significantly reduced adverse events compared with PTBD (RR, 0.37; 95% CI, 0.14–0.97) [8]. This discrepancy may be the result of EUS-HGS, which is technically more demanding than EUS-CDS, leading to more complications, whereas RCTs primarily utilized EUS-CDS as a preferred method. When considering PTBD outcomes, our study observed no major complications, whereas in some studies the complication rate exceeded 30–40% in prior PTBD series [9].

Comparing our results to a multicenter Japanese analysis by Itonaga et al. where patients with EUS-BD and PTBD to treat malignant distal bile duct obstruction (MDBO) after a failed ERCP went on to pancreaticoduodenectomy (PD), we conclude that both methods seemed effective for MBO management in terms of technical and early biochemical response when done in expert centers. Unlike our largely palliative MBO cohort, their focus was mostly on postoperative outcomes, such as DFS and OS. Itonaga et al. reported no effect of drainage type on postoperative adverse events, which were instead linked to age and ASA status, but we noticed increased drainage-related and significant problems following EUS-BD, mostly due to the complicated EUS-HGS procedure. Significantly, their research demonstrated the procedural benefits of EUS-BD, such as increased internalization, fewer sessions, and a shorter hospital stay [10].

### 4.3. Clinical Implications

Our findings have some implications for clinical decision-making in MBO after a failed ERCP. First, EUS-BD and PTBD are equally effective in terms of technical success and early biochemical response, proving that either modality can serve as an effective rescue drainage strategy when performed by experienced specialists. However, the EUS-BD group was burdened with a higher complication rate, especially EUS-HGS. In particular, the occurrence of major Clavien–Dindo ≥ III complications exclusively in the EUS-BD cohort vs. PTBD (10.8% vs. 0%) implies that patient safety profiles differ meaningfully between these two options. Clinically, this finding highlights the need for careful patient selection when considering EUS-BD, especially in settings where complex procedures such as hepaticogastrostomy may be required.

Despite the higher complication burden, EUS-BD was associated with a longer observed OS compared with PTBD (median 143 vs. 54 days and RMST difference + 39.6 days at 180 days), indicating that earlier physiological benefits, improved internal biliary drainage, or reduced long-term device-related morbidity may translate into observed survival advantages. However, the underlying reasons for this association remain uncertain and may reflect differences in patient selection, disease burden, anatomical feasibility, and supportive care rather than the drainage technique itself.

Not all EUS-BD techniques are equally safe. While EUS-CDS offered great efficacy with low complication rates, EUS-HGS was associated with much higher complication rates, especially severe ones. Based on this, the best approach would be holistic, with recognition of the anatomy of the biliary tree, tumor location, operators’ expertise and experience, and technical possibility to approach as EUS-CDS. Also, PTBD may be a safer alternative in such situations. It is worth mentioning that the PTBD group in our study did exceptionally well, receiving low complication rates, especially severe complications that did not occur in our cohort. This supports the statement that PTBD can be a preferred option for unstable patients with altered anatomy and in circumstances where EUS is not technically feasible.

However, both methods of drainage for MBO are associated with the presence of early and late complications. EUS-BD carries a non-negligible risk of adverse events, most commonly stent misdeployment, cholangitis, bleeding, bile leak, and stent obstruction, although the majority are mild to moderate when performed by experienced endoscopists. However, serious events such as perforation or peritonitis can occur, particularly with more complex cases [11]. Percutaneous biliary drainage also carries several specific risks, including catheter-related problems such as occlusion, dislodgement, fracture, or bile leakage, which may lead to local skin injury or biloma formation. Vascular complications, such as arterial injury, pseudoaneurysm, hemobilia, or venous fistula, and infectious events, including cholangitis, sepsis, or hepatic abscess, are also recognized. Rare but serious complications include pneumothorax/hemothorax from a pleural puncture, bowel injury, or tumor tract seeding [12].

### 4.4. Initial Condition of Patients in Both Cohorts

Although patients in the PTBD cohort initially appeared to be in a worse clinical state, an impression driven largely by higher baseline bilirubin level, the comprehensive comparison of inflammatory markers and composite indices did not support the difference in the baseline systematic disease burden between groups. Except for baseline total bilirubin, none of the analyzed laboratory parameters (CRP, platelets, NLR, LMR, PLR, SII, SIRI, NPS, and LCR) differed significantly. This suggests that the two groups were similar with respect to the measured inflammatory markers, although unmeasured or underreported clinical factors such as frailty, performance status, tumor extent, or anatomical complexity may still have differed and influenced treatment allocation. While the PTBD group may have seemed clinically more affected by jaundice at presentation, statistical analysis did not confirm any major imbalance between both groups in terms of initial MBO severity.

Recent meta-analyses and large cohort studies consistently demonstrate that the NLR is the most robust and independent prognostic marker for the OS in pancreatic ductal adenocarcinoma (PDAC), both in resectable and advanced/metastatic settings [13,14,15,16,17,18]. Elevated NLR is associated with significantly poorer survival, with optimal cutoffs typically ranging from 3.3 to 5 depending on the cohort and disease stage [16,17,18].

PLR also predicts worse survival, but its prognostic strength is less consistent and often does not remain significant in multivariate models when compared directly to NLR [13,14,17,19,20,21,22]. PLR may be useful in combination with NLR or other markers for improved risk stratification [21].

LMR is an independent prognostic factor in resectable, locally advanced, and metastatic PDAC, with lower values correlating with reduced survival. A cut-off of four has been shown to identify patients with significantly better outcomes in metastatic disease [23,24].

SII, calculated as (platelet count × neutrophil count)/lymphocyte count, is associated with poor survival in PDAC, with meta-analyses confirming its prognostic value for both short- and long-term outcomes [25,26]. However, SII does not outperform NLR in most direct comparisons.

LCR and the related CLR are emerging markers. Low LCR and high CLR are independently associated with poor survival and recurrence, and CLR may outperform NLR and PLR in some analyses [27,28].

In summary, NLR provides the highest prognostic accuracy among these indices, followed by LMR and SII, with PLR and LCR/CLR offering an additional but less consistent value. Combining NLR with other markers (e.g., PLR and cfDNA) may further enhance prognostic stratification in PDAC.

The pathophysiological mechanism underlying the prognostic value of peripheral blood inflammatory indices in PDAC is driven by the interplay between systemic inflammation and tumor-induced immunosuppression [29]. Elevated NLR, PLR, and low LMR reflect a shift toward a pro-tumor inflammatory state and a reduction in anti-tumor immune surveillance.

High neutrophil counts indicate increased release of pro-inflammatory cytokines and growth factors (e.g., vascular endothelial growth factor, VEGF and interleukin-6, IL-6) that promote tumor proliferation, angiogenesis, and metastasis. Neutrophils also suppress cytotoxic lymphocyte activity, further impairing anti-tumor immunity. Conversely, low lymphocyte counts signal a reduced adaptive immune response, particularly diminishing populations of helper and cytotoxic T cells, B cells, and natural killer (NK) cells, which are critical for tumor cell recognition and destruction.

Elevated monocyte counts (reflected in low LMR) are associated with increased tumor-associated macrophages, which facilitate tumor progression and immune evasion. Platelets contribute to tumor growth and dissemination by shielding circulating tumor cells from immune attacks and by promoting extravasation and angiogenesis, explaining the prognostic value of PLR. Indices such as the SI and LCR integrate these cellular and protein markers, further capturing the balance between inflammation and immune competence.

Collectively, these indices serve as surrogates for the degree of systemic inflammation and immune dysfunction, which are central to PDAC progression and poor outcomes. However, these markers should be interpreted with caution when assessing baseline comparability. While such indices have been associated with disease severity and prognosis in hepatobiliary and pancreatic malignancies, they are not routinely used in clinical decision-making globally and cannot substitute for comprehensive clinical assessment. In this study, these parameters were therefore included as exploratory, hypothesis-generating measures rather than as definitive indicators of equivalence between cohorts. The absence of statistically significant differences in these indices does not exclude clinically meaningful differences in functional status, anesthetic risk, tumor anatomy, or overall frailty-factors that likely influence the non-random allocation to EUS-guided or percutaneous drainage. Accordingly, these laboratory markers should be viewed as complementary contextual data rather than proof of a comparable baseline health status between treatment groups.

Also, the difference in baseline bilirubin concentration may be due to technical limitations of the methods. PTBD becomes technically feasible when the hepatic or segmental bile ducts are at least 8 mm wide; this usually makes PTBD patients wait for a few more days in the hospital before the actual procedure. In EUS-BD (especially GBD), the widening of bile ducts is not as important as PTBD to make the procedure technically feasible, as the main goal of the procedure is to restore the natural bile flow into the digestive tract. Only EUS-HGS may require wide segmental bile ducts to be approximately ~4 mm to make it technically possible to restore biliary flow [7]. Also, in EUS-CDS, a CBD diameter ≥ 13 mm is required.

EUS-HGS and EUS-GBD were performed only in cases where EUS-CDS was technically unfeasible due to anatomico-oncological difficulties. This distinction is clinically essential, as current international guidelines consistently identify EUS-CDS as the preferred and most standardized EUS-guided drainage technique due to its high technical success, favorable safety profile, and lower procedural complexity [30,31,32]. Consequently, cases in which EUS-CDS is not feasible represent a more advanced oncologic stage, often with a greater local tumor burden, a more extensive duodenal involvement, and a poorer baseline prognosis. In our cohort, patients undergoing EUS-HGS or EUS-GBD carried a higher procedural risk, an increased likelihood of adverse events, and a generally worse expected survival irrespective of drainage modality. Therefore, the higher complication rates observed in the EUS-HGS and EUS-GBD subgroups in our cohort should not be interpreted as equivalent procedural alternatives to EUS-CDS, but rather as salvage interventions used only when the guideline-preferred method is anatomically impossible.

Quality of life after the procedures also remains an important issue for consideration. EUS-BD patients have preserved anatomical flow of bile to the digestive tract. This continuity enables effective emulsification and absorption of dietary lipids, thereby supporting normal digestion and maintaining adequate levels of fat-soluble vitamins, including vitamins A, D_3_, E, and K. Preservation of enteral bile flow also contributes to overall nutritional status and metabolic homeostasis.

In contrast, PTBD patients may suffer from steatorrhea, vitamin deficiencies, and malnutrition/weight loss/cachexia, and a subsequent decline in overall quality of life. A lack of vitamins causes osteopenia, osteoporosis, easy bruising, bleeding, muscle weakness, and worse eyesight at night. Moreover, external biliary drainage is associated with additional practical and psycho-social burdens. The necessity for continuous drainage of bile with the use of syringes and drainage bags requires regular emptying, handling, and monitoring, which can be uncomfortable and inconvenient. External drains can interfere with daily activities, personal hygiene, and sleep, posing a risk of accidental displacement of the drain, bile leakage, skin irritation, and infection at the catheter insertion site. These factors might negatively affect patients’ autonomy, body image, and overall well-being. Internal drainage, such as EUS-BD, is less of a burden in terms of quality of life. However, it should be recognized that palliative patients still achieve a significant benefit in terms of reducing symptoms such as pruritus or jaundice, regardless of the drainage method; thus, their quality of life is generally better [33,34].

### 4.5. Limitations

This study has several limitations. First, its single-center, retrospective design and non-randomized allocation based on operational availability introduce susceptibility to confounding and selection bias, despite the absence of explicit clinician or patient preference for one modality. Second, EUS-BD was implemented later than PTBD; therefore, calendar time and evolving supportive care may confound survival comparisons even after complementing log-rank tests with RMST over a common horizon. Third, the overall sample size, particularly within procedure subtypes and for major complications (Clavien–Dindo ≥ III), limits statistical power. Fourth, we defined early biochemical response as a ≥5% bilirubin reduction by day two to capture very early efficacy. This early window may differ from definitions used elsewhere (e.g., on day 14 in Tokyo guidelines) and could underestimate delayed biochemical responses. Fifth, we did not compare patient-reported outcomes or quality of life between the procedures.

Although the EUS-BD cohort included different technical approaches (EUS-CDS, EUS-HGS, and EUS-GBD), this heterogeneity reflects real-world anatomical and oncologic constraints rather than discretionary variability. Importantly, the underlying disease was largely homogeneous, as approximately 90% of patients had pancreatic head cancer causing malignant biliary obstruction. EUS-CDS, the guideline-preferred technique in distant MBO, was used whenever technically feasible, whereas alternative approaches were reserved for cases with advanced local disease or unfavorable anatomy. These salvage techniques represent more complex clinical scenarios and may partly explain the higher complication rates observed within the EUS-BD group. Moreover, because effective biliary drainage is a prerequisite for initiating systemic therapy, and standardized data on post-drainage oncologic treatment were not available, the observed survival differences should be interpreted cautiously, as they may reflect differences in subsequent systemic therapy rather than a direct effect of the drainage modality.

It is plausible that patients selected for EUS-guided biliary drainage represented a clinically healthier (less burdened by disease) subgroup, which could partially explain the observed survival advantage. In routine practice, eligibility for EUS-BD depends on anesthetic tolerance and favorable anatomical conditions, which are factors that may preferentially select patients with better functional reserve. Although baseline inflammatory markers and composite indices did not differ significantly between groups, these parameters cannot fully capture differences in frailty, performance status, or tumor-related anatomical complexity. Therefore, the longer survival observed after EUS-BD should be interpreted strictly as an association, which may reflect patient selection, temporal bias, and unmeasured confounders, and cannot be used to infer superiority or causality of EUS-BD over PTBD. Although consecutive inclusion and non-preferential operational allocation mitigate overt selection, residual confounding by indication and calendar time remains possible in this retrospective setting.

### 4.6. Future Directions and Need for Further Research

The lack of clarity on the biological and clinical variables that contribute to the survival difference between EUS-BD and PTBD, despite comparable success in early bilirubin reduction, is a major gap in understanding that our work emphasizes. Moreover, the study cannot specify which patients, based on tumor features, architecture, or inflammatory status, can benefit the most from a given draining approach. The specific impacts of individual EUS-BD techniques (CDS/HGS/GBD) and PTBD sites (LHD/RHD/GB/CBD) on oncologic trajectories, quality of life, and integration with systemic medicines remain unknown.

To sum up, our findings support a balanced, patient-tailored approach to biliary drainage after a failed ERCP. Given the retrospective, single-center design, non-standardized allocation between EUS-BD and PTBD, and the potential for selection bias, these results should be considered hypothesis-generating. Well-designed, adequately powered, multicenter randomized controlled trials comparing EUS-BD and PTBD are needed to clarify their relative safety, efficacy, impact on life quality, and survival outcomes. While EUS-BD might offer superior long-term outcomes, positively affecting OS, as its higher complication rates can make this technique unfeasible for some patients. A multidisciplinary discussion involving surgeons, endoscopists, interventional radiologists, and oncologists remains essential to tailoring the drainage strategy to individual patient needs.

## 5. Conclusions

In this high-volume single-center cohort of patients with MBO after a failed ERCP, both EUS-BD and PTBD performed well in terms of technical success and had a comparably early biochemical response. However, EUS-BD was associated with a higher overall complication rate, including severe complications. On the other hand, EUS-BD was associated with longer observed overall survival; however, this finding should be interpreted with caution, as it may be influenced by patient selection, anatomical feasibility, and the retrospective, non-randomized nature of the study. These findings support the use of both modalities as an effective rescue therapy in MBO, emphasizing the need for careful patient selection and careful planning of the procedure by including the anatomy of the biliary tree and the location of the tumor. The decision about the most feasible method of drainage should be made by a multidisciplinary team consisting of specialists in surgery, oncology, gastroenterology, and radiology. Prospective, multicenter randomized controlled trials are required to determine whether EUS-BD offers a true survival advantage over PTBD and to define the optimal drainage strategy for specific patient subgroups. Because this was a single-center retrospective study with non-standardized allocation between EUS-BD and PTBD, our findings should be interpreted as exploratory and hypothesis-generating.

## Figures and Tables

**Table 1:** General characteristics of the study groups. Nominal data are presented as *n* (%); continuous data are presented as median (IQR). *p*-values for continuous variables are from two-sided Mann–Whitney U tests; counts are descriptive. Among the variables compared, only baseline total bilirubin differed significantly between groups (EUS vs. PTBD, *p* = 0.0012).

Characteristic/Marker	EUS-BD	PTBD	*p*-Value
Sample size	37	64	—
Age	70.0 (66.0–76.0)	68.5 (61.2–72.2)	—
Female/Male	24/13	37/27	—
CRP (mg/L)	37.7 (18–158)	55.3 (23.9–111)	0.795
PLT (×10^9^/L)	237 (205–325)	292 (210–367)	0.206
NLR	5.64 (3.71–7.48)	6.46 (2.87–9.42)	0.889
PLR	209 (137–307)	220 (157–311)	0.624
LMR	1.92 (1.27–2.46)	2.00 (1.18–3.00)	0.955
SII	1.32 × 10^3^ (0.888–1.93 × 10^3^)	1.43 × 10^3^ (0.718–3.74 × 10^3^)	0.864
SIRI	3.29 (2.33–5.07)	3.23 (1.71–7.10)	0.858
NPS	0 (0–1)	0 (0–1)	0.603
LCR	0.0286 (0.0113–0.0832)	0.0213 (0.0095–0.0616)	0.505
Total bilirubin (baseline)	10.5 (5.59–19.1)	16.5 (12.8–23.3)	0.001

Abbreviations: EUS-BD—endoscopic ultrasound-guided biliary drainage; PTBD—percutaneous transhepatic biliary drainage; CRP—C-reactive protein; PLT—platelets; NLR—neutrophil-to-lymphocyte ratio; PLR—platelet-to-lymphocyte ratio; LMR—lymphocyte-to-monocyte ratio; SII—systemic immune-inflammation index; SIRI—systemic inflammation response index; NPS—neutrophil-to-platelet score; LCR—lymphocyte-to-CRP ratio; and IQR—interquartile range.

**Table 2:** Nominal outcomes are shown as *n*/N (%) and continuous outcomes as median (IQR). Overall, EUS–PTBD comparisons use Fisher’s exact test for binary variables and the Mann–Whitney U test for the LOS; within-method comparisons across subtypes use the chi-square/Fisher (binary) test and Kruskal–Wallis test (LOS). Technical success: since both methods achieved 100% with no failures, the between-group test is n/a (uninformative with zero variance). If desired, exact 95% Clopper–Pearson CIs can be reported alongside the 100% proportions to reflect sample size. NA indicates that testing was not applicable due to zero events in relevant cells.

Section/Subtype	N	Technical Success	Early Biochemical Response ≥ 5% (D+2)	Any Complication	Major Complication (Clavien ≥ III)	LOS, Days
EUS-BD	37	37/37 (100%)	32/37 (86.5%)	11/37 (29.7%)	4/37 (10.8%)	4.0 (2.0–7.0)
PTBD	64	64/64 (100%)	50/64 (78.1%)	8/64 (12.5%)	0/64 (0.0%)	3.5 (2.0–6.0)
*p*-value	—	n/a	0.43	0.04	0.02	0.21
**EUS-BD subtypes**						
GBD	6	6/6 (100%)	4/6 (66.7%)	1/6 (16.7%)	0/1 (0.0%)	7.0 (6.2–8.5)
CDS	21	21/21 (100%)	21/21 (100.0%)	4/21 (19.0%)	1/4 (25.0%)	3.0 (2.0–6.0)
HGS	10	10/10 (100%)	7/10 (70.0%)	6/10 (60.0%)	3/6 (50.0%)	5.0 (2.2–13.2)
*p*-value	—	—	0.02	0.049	0.53	0.28
**PTBD subtypes**						
LHD	40	40/40 (100%)	31/40 (77.5%)	6/40 (15.0%)	0/6 (0.0%)	4.0 (1.8–6.0)
RHD	1	1/1 (100%)	0/1 (0.0%)	0/1 (0.0%)	n/a	9.0 (9.0–9.0)
GB	20	20/20 (100%)	16/20 (80.0%)	2/20 (10.0%)	0/2 (0.0%)	3.0 (1.8–5.2)
CBD	3	3/3 (100%)	3/3 (100.0%)	0/3 (0.0%)	n/a	8.0 (5.0–8.0)
*p*-value	—	—	0.38	0.17	n/a	0.15

Abbreviations: EUS-BD—endoscopic ultrasound-guided biliary drainage; PTBD—percutaneous transhepatic biliary drainage; CDS—choledochoduodenostomy; HGS—hepaticogastrostomy; GBD—EUS-guided gallbladder drainage; LHD—left hepatic duct drainage; RHD—right hepatic duct drainage; GB—gallbladder drainage; CBD—common hepatic/common bile duct drainage; LOS—length of stay; and IQR—interquartile range.

**Table 3:** Procedure-related adverse events (AEs) by treatment arm and typical management. Values are shown as *n*/N (%) with arm-specific denominators (EUS N = 37 and PTBD N = 64). Categories include bleeding/anemization (including hemobilia and periliver hematoma), biloma/post-drain fluid collection, and gastrointestinal perforation; the composite index “Any AE” reflects patients with ≥1 event. Severity is summarized by the worst Clavien–Dindo grade per patient (EUS: II 18.9%, IIIa 5.4%, V 5.4% and PTBD: II 12.5%, ≥III 0%). Typical management pathways are indicated (conservative ± transfusion; endoscopic/radiologic intervention; percutaneous drainage; and surgical repair) and reflect the actions taken when required. Abbreviations: AE—adverse event; EUS—endoscopic ultrasound-guided biliary drainage; PTBD—percutaneous transhepatic biliary drainage.

AE Category	EUS (*n*, %)	PTBD (*n*, %)	Typical Management
Bleeding/anemization (incl. hemobilia, periliver hematoma)	8/37 (21.6%)	8/64 (12.5%)	Conservative measures ± transfusion; endoscopic/radiologic hemostasis if needed
Biloma/post-drain fluid collection	1/37 (2.7%)	0/64 (0.0%)	Percutaneous drainage and/or device revision
Gastrointestinal perforation	2/37 (5.4%)	0/64 (0.0%)	Endoscopic, radiologic, or surgical repair
Any AE (≥Clavien I)	11/37 (29.7%)	8/64 (12.5%)	—

**Table 4:** Numbers at risk at prespecified landmarks with percentages relative to the baseline size of each arm. This presentation helps readers gauge follow-up density at each time point (e.g., ≥10–20% are still at risk at 180 days in both groups), supporting the choice of τ = 180 for RMST.

Group (Baseline *n*)	0 d	30 d	90 d	180 d	360 d
EUS-BD (*n* = 37)	37 (100%)	26 (70.3%)	15 (40.5%)	8 (21.6%)	2 (5.4%)
PTBD (*n* = 64)	64 (100%)	42 (65.6%)	20 (31.3%)	8 (12.5%)	2 (3.1%)

## Data Availability

The data analyzed can be given upon reasonable request.
